# Speech perception and quality of life of open-fit hearing aid users

**DOI:** 10.1590/1678-775720150321

**Published:** 2016

**Authors:** Tatiana Manfrini GARCIA, Regina Tangerino de Souza JACOB, Maria Fernanda Capoani Garcia MONDELLI

**Affiliations:** Universidade de São Paulo, Faculdade de Odontologia de Bauru, Departamento de Fonoaudiologia, Bauru, SP, Brasil.

**Keywords:** Hearing aids, Hearing loss, Quality of life, Speech perception

## Abstract

**Objective:**

To relate the performance of individuals with hearing loss at high frequencies in speech perception with the quality of life before and after the fitting of an open-fit hearing aid (HA).

**Methods:**

The WHOQOL-BREF had been used before the fitting and 90 days after the use of HA. The Hearing in Noise Test (HINT) had been conducted in two phases: (1) at the time of fitting without an HA (situation A) and with an HA (situation B); (2) with an HA 90 days after fitting (situation C).

**Study Sample:**

Thirty subjects with sensorineural hearing loss at high frequencies.

**Results:**

By using an analysis of variance and the Tukey’s test comparing the three HINT situations in quiet and noisy environments, an improvement has been observed after the HA fitting. The results of the WHOQOL-BREF have showed an improvement in the quality of life after the HA fitting (paired t-test). The relationship between speech perception and quality of life before the HA fitting indicated a significant relationship between speech recognition in noisy environments and in the domain of social relations after the HA fitting (Pearson’s correlation coefficient).

**Conclusions:**

The auditory stimulation has improved speech perception and the quality of life of individuals.

## INTRODUCTION

Hearing aid users show difficulty in perceiving speech when in the presence of competing background noise, which may affect their social interaction. The auditory deprivation causes consequences in the individual’s life and affects the ability to properly understand acoustic information, as well as in the way they relate to their environment, which can cause a decisive impact on their quality of life (QoL).

Individuals who exhibit hearing loss restricted to high frequencies (above 1 kHz) in adverse conditions, such as when speech is distorted, or in the presence of noise, may face several difficulties in speech intelligibility because the number of auditory cues drops considerably. The speech intelligibility depends on the consonant sounds that present sound spectrum with frequencies above 2 kHz. The fact that the consonants are low intensity sounds in relation to vowels makes them more difficult to detect, especially for individuals with slope hearing loss^9^. The purpose of amplification in individuals with this audiological profile is to provide an emphasis on high frequencies sounds, providing audibility of speech signal without generating acoustic feedback or distortion, and avoiding autophonia from the occlusion of the external auditory canal (EAC) by the earmold. The total or partial occlusion of the EAC with an earmold causes the loss of natural resonance of the EAC, which makes it difficult to obtain gains in the region of 3–4 kHz. Therefore, some strategies should be considered during the hearing aids (HAs) selection such as the open-fit (OF) HAs^8^.

The OF is a specific miniature behind the ear (BTE - HA) that uses a thin sound tube and a soft vented silicone eartip holding the tube in place inside the canal without using an earmold. Open-fit does not only refer to the HA model, but also to the EAC condition not being occluded, which can be verified through measurements with probe microphone real-ear occluded response (REOR) equal to the real-ear unaided response (REUR) allowing the use of the term “open-fit” throughout the article.

One way to measure success in the HAs fitting is to assess the improvement in speech recognition, particularly in noisy environments, which could possibly lead to an improvement in the individual QoL.

Over the years, many tests of speech perception in noise have been developed in an attempt to better evaluate the individual’s performance in noisy environments, seeking to maximize the approach in situations of daily life such as CTS - Connected Speech Test, SIN - Speech in Noise Test, QuickSIN-QuickSpeech-in-Noise Test, BKB-SIN - Bamford-Kowal-Bench Speech-in-Noise Test, SPIN - Speech Perception In Noise, and HINT – Hearing in Noise Test. There are differences between the tests in the speech materials that influence the assessment of speech intelligibility in noisy environments^15^. Tests that contain sentences in noisy environments, such as HINT, represent everyday speech and are more effective in the measurement of difficulties in understanding speech by exposing the individual to different variations in the signal-to-noise ratio (SNR) in each environment^13^. The HINT is widely used to objectively demonstrate the benefit of HAs, evaluate and verify HAs algorithms such as directional microphones, expansion, noise reduction, wireless and various other models of HAs^5^ ^,^ ^7^ ^,^ ^11^ ^,^ ^14^ ^,^ ^18^ ^-^ ^21^.

Literature shows that the verification and validation in the fitting of HAs decreased the number of returns and increased the patient’s satisfaction with HAs. Thus, the author suggested perception of speech in noise tests for validation^6^.

Several studies point to a negative assessment of QoL in individuals with hearing loss, but few studies have used specific instruments to measure the QoL, such as the WHOQOL-Breef, related to hearing loss. These surveys are scarce in the literature, which makes this topic a necessary study subject in the audiology area^2^.

Slope hearing loss configurations are prevalent in audiological clinics. Subjects with the slope hearing loss do not often feel hearing difficulties in the silence, thus they do not present hearing complaints, since they have normal hearing up to 1 kHz and moderate loss to high tones, which causes difficulty only in noisy environments. The indication of HA for this population should be based on the difficulty presented in noise. The speech perception test is an important tool that can objectively show the improvement for the patients, therefore improving their quality of life.

Studies on the QoL and the speech perception of individuals with hearing loss at high frequencies may fundamentally contribute to the field of audiology, aiding and/or confirming the decision-making process regarding the prescription of HAs and providing more support for the counseling and guidance processes in the use of OF fitting.

The aim of this study was to analyze the relationship between speech perception and QoL in adults and elderly with hearing loss at high frequencies, before and after the adaptation of OF HAs.

## METHODS

This prospective cohort study has been conducted after approval by the Ethics Committee for Research in Humans (607.178) and after the agreement and informed consent of the subjects.

The study included 30 individuals who have met the following inclusion criteria:

Aged more than 30 years;

Diagnosed with sensorineural hearing loss with a slope hearing loss, restricted at high frequencies (above 1 kHz), compatible with a conventional OF HA (Receiver in the advice - RITA) fitting, and with digital technology;

No prior experience with the use of HAs;

Absence of other pathology associated with hearing loss;

Ability to understand the WHOQOL-BREF questionnaire. If subjects had any difficulties in answering the questions, they would be excluded from the sample.

### Participants

**Figure 1:**
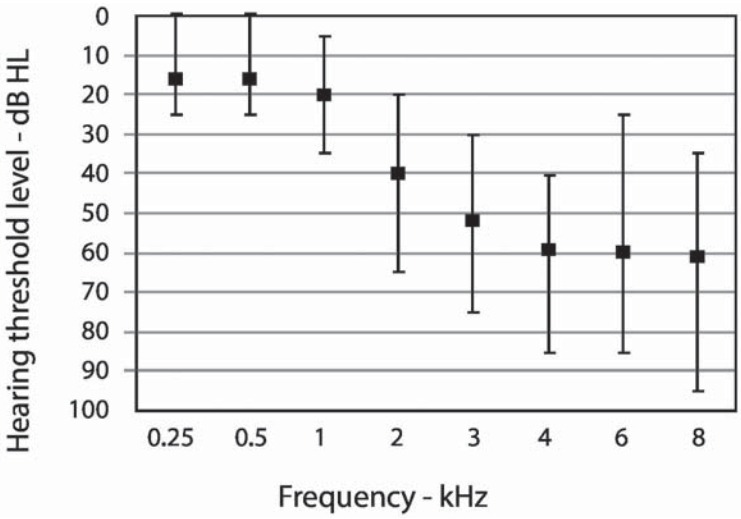
Averages, minimum and maximum audiometric thresholds

The sample consisted of 11 female and 19 male aging from 34 to 78 years (mean: 61, 41 sd: 9, 67). The audiometric threshold averages are in Figure 1.

### Procedures

#### Selection and verification of HAs

In order to select the type and model of HA, the audiological features and communicative needs of the participants have been analyzed. According to this analysis, the OF HAs were selected. The HAs selected was the miniature digital BTE type, with four channels, a directional microphone system, digital noise reduction, and feedback cancellation by reversed phase technology. All HAs were the same manufacturer and model.

The HAs have been programmed via HI-Pro and the participant’s identification data, such as name, date of birth, gender, and audiometric thresholds, have been input into the NOAH platform version 3.0 (HIMSA - Copenhagen, Denmark).

When programming the enterprise software, the level of user experience has been defined as “experienced”, with the aim to reach target. Information related to the acoustic characteristics of the thin tube and probes have been added. The NAL-NL1 prescriptive method has been used based on the previously entered thresholds. The algorithms for digital noise reduction and directional microphones have been activated for all participants.

After programming the HAs, a verification procedure using measurements from a probe microphone has been performed. This procedure has been performed in an acoustically treated room using Affinity 2.0 equipment (Interacoustics – Middelfart, Denmark). The following measurements have then been performed: real-ear unaided response (REUR), open-fit calibration, real-ear occluded response (REOR) always equal to the real-ear unaided response (REUR), and real-ear aided response (REAR).

The values of the REAR at frequencies from 0.25 to 6 kHz for input levels of 50, 65, and 80 dB SPL have been respectively compared with the NAL-NL1 targets. Responses were considered equivalent when the difference between the target and the REAR value did not exceed 10 dB^3^. The responses have reached targets for all subjects.

## Evaluation of speech perception

The assessment of speech perception has been performed using the HINT adapted to Brazilian Portuguese^1^ under two conditions:

Sentences presented in a quiet environment (quiet);

Sentences presented in competitive noise; the type used was masking composite noise, with only the frontal position noise being used (noise front);

In both conditions the sentences have been presented first unaided and then aided.

The HINT test have been performed in three situations and divided into two phases as follows:

Phase 1: at the time of HA fitting in situations unaided (situation A) and aided (situation B).

Phase 2: aided 90 days after the HA fitting (situation C).

The test has been conducted in an acoustically treated room, using free-field sentences. In all evaluation conditions, the signal (speech and noise) was presented from a single speaker at 0° azimuth, 1 m from the listener at a height of the head. The system calibration has been performed by placing a microphone at the reference corresponding to the participant’s head center location and 1 m away from the speaker.

A list of 20 sentences has been randomly presented by the HINT PRO software for each condition. Participants have been instructed orally as to the guidelines contained in the HINT manual.

The sentence was considered correct by the measurer when all essential words were repeated correctly. In this case, the examiner pressed the “yes” button on the software screen, and the next sentence was presented at 2 dB below the intensity of the previous sentence.

For the sentences in quiet condition, the presentation level was initially set at 45 dB(A). The presentation level was increased in steps of 2 dB until the participant correctly repeated the sentence. The score for this test was expressed as dB(A) level, at which the participant correctly repeated 50% of the sentences.

The level of noise was fixed at 65 dB(A) for the sentences and the presentation level was initially set at 65 dB(A). The level of presentation of the sentences varied in the same manner as described for the sentences in quiet condition. The score was expressed in dB as being the SNR after the presentation of a list of 20 sentences. Therefore, the lower the SNR is, the better the speech perception for the participant under this condition.

## Assessment of QoL

The WHOQOL-BREF questionnaire was used to assess different aspects of QoL of the participants. This questionnaire is an abbreviated version of the WHOQOL-100 developed by the World Health Organization and validated in Brazil by Fleck, et al.^4^ (2000).

The WHOQOL-BREF consists of 26 questions with two pertaining to general QoL issues and the other 24 questions representing each of the 24 facets that compose the original instrument (WHOQOL-100). Thus, the 24 questions cover four domains (physical, psychological, social relationships, and environment) and each facet is represented by a question^5^. The WHOQOL-BREF domains are:

1. Physical domain: corresponding to issues related to pain, discomfort, energy, fatigue, sleep, resting, mobility, activities of daily living, dependence on medication or treatment, and the ability to work.
2. Psychological domain: corresponding to questions about positive feelings, thinking, learning, memory and concentration, self-esteem, body image and appearance, negative feelings, spirituality, religion, and personal beliefs.
3. Environment domain: corresponding to questions about physical safety and protection, home environment, finances, health care, social care, accessibility and quality of opportunities to acquire information and skills, opportunities and participation in recreation and leisure, the physical environment (pollution/noise/traffic/climate), and transportation.
4. Social relations domain: corresponding to questions about personal relationships, social support, and sexual activity.

The WHOQOL-BREF includes four types of response scales: intensity (ranging from nothing to intense); capacity (ranging from none to full); frequency (ranging from never to always); and evaluation (ranging from very dissatisfied to very satisfied and very bad to very good), all being graded in five levels. The questions are scored from 1 to 5, and the scores are reversed for questions 3, 4, and 26, in such a way that 1=5; 2=4; 3=3; 4=2; and 5=1.

After the questionnaire had been completed, general and domain (physical, psychological, environmental, and social relations) values were calculated, allowing for an evaluation of the individual QoL. This analysis has been performed according to the syntax described by the translators using the Statistical Package for Social Science (SPSS) 10.0 for Windows.

The participants completed the questionnaire before the fitting of the HAs and after 90 days of the fitting.

## Statistical analysis

The results have been analyzed using descriptive and inductive statistical analysis. All statistical procedures have been performed on STATISTICA software version 5.1 (StatSoft, Inc., Tulsa, USA). The level of significance had been set at 5% in all cases.

In the comparison of speech perception (HINT) at the time of fitting in situations without an HA, with HAs, and with an HA post- fitting, analysis of variance and Tukey’s test have been applied. Comparisons of the results of the WHOQOL-BREF unaided and aided have been performed using paired t-tests. The Pearson’s correlation test has been applied to determine whether there has been a correlation between speech perception and QoL scores in the different domains of the WHOQOL-BREF.

## RESULTS

The results of the HINT at the time of fitting in situations unaided (situation A), aided (situation B), and aided 90 days after HA fitting (situation C) are shown in Table 1.

**Table 1:** Values of the responses obtained in the Hearing in Noise Test in quiet and noisy environments

	QUIET			NOISY		
	HINT A	HINT B	HINT C	HINT A	HINT B	HINT C
Mean	43.36	38.05	39.72	1.01	-0.51	-1.1
Mediam	41.35	37.6	38.4	0.55	-0.2	-1.8
Minimum	33.5	30.6	30.8	-3	-4	-4.1
Maximum	57.8	50	57.5	10.6	3	4
SD	7.15	5.46	5.78	2.91	1.74	2.07

A= unaided; B= aided; C= aided 90 days after the HA fitting

The analysis of variance and Tukey’s test comparing the three HINT situations under quiet and noisy conditions have showed significant improvements comparing situation A to B (0.00012) and A to C (0.00076), but there has been no difference between situations B and C (0.17659).

The results of the WHOQOL-BREF unaided and aided are described in Table 2.

**Table 2:** Values obtained when applying the World Health Organization Quality of Life (WHOQOL-bref) instrument unaided and aided

	Domains	Mean	SD	CV	Minimum	Maximum
Unaided	P	14.27	2.98	20.92	5.71	18.86
	Ps	14.82	2.23	15.02	9.33	18.67
	SR	14.44	3.32	23.01	4	20
	E	13.8	2.59	18.73	6.5	19
	SR/QL	14.87	2.66	17.9	8	20
	Total	14.32	2.16	15.08	8.77	18.46

Aided	P	15.45	2.52	16.3	10.86	20
	Ps	16.49	1.66	10.07	13.33	20
	SR	16.67	2.34	14.05	10.67	20
	E	15.55	1.83	11.77	12	19.5
	SR/QL	16.93	2.27	13.42	12	20
	Total	15.97	1.62	10.13	13.38	19.38

P=physical; Ps=psychological; SR=social relationships; E=environment; SR/QL=self assessment/quality of life

Comparisons of the results of the WHOQOL-BREF in situations A and C have showed significant improvements in all domains by paired t-tests (p>0.005), with the largest difference in the social relations domain, followed by the self-assessment of QoL domain.

The relationship between speech perception and QoL in situations A and C has been performed using Pearson’s correlation coefficient, and the values of p and r are described in Table 3.

**Table 3:** Values p and r in Pearson correlation between World Health Organization Quality of Life (WHOQOL-bref) and Hearing in Noise Test unaided and aided

		WHOQOL-bref domains					
		P	Ps	SR	E	SR/QL	Total
Unaided	HINT Q	r=0.29	r=0.12	r=0.29	r=0.16	r=0.24	r=0.27
		p=0.11	p=0.50	p=0.11	p=0.38	p=0.18	p=0.13
	HINT NF	r=0.26	r=-0.00	r=0.25	r=0.21	r=0.35	r=0.25
		p=0.16	p=0.96	p=0.17	p=0.25	p=0.05	p=0.17

Aided	HINT Q	r=0.22	r=0.16	r=0.21	r=-0.02	r=0.19	r=0.18
		p=0.23	p=0.39	p=0.25	p=0.90	p=0.32	p=0.34
	HINT NF	r=0.24	r=0.33	r=0.63	r=0.11	r=0.13	r=0.33
		p=0.20	p=0.07	p=0.00	p=0.57	p=0.48	p=0.07

P=physical; Ps=psychological; SR=social relationships; E=environment; SR/QL=self assessment/quality of life; Q=quiet; NF=noise front

## DISCUSSION

The HINT has been used as an outcome measurement for HAs. The mean threshold for speech recognition in quiet conditions aided (situation B) was 5.31 dB lower than unaided (situation A). Comparing the results after 90 days of fitting to the results without an HA (situation C and A), the difference was 3.74 dB. These differences were statistically significant (p=0.00012; p=0.00076) and demonstrated that amplification provides better speech perception in quiet^11^.

In relation to the performance in speech perception in noise, the SNR scores comparing first situations A and B, then situations A and C were respectively 0.5 dB and 2.11 dB lower. These differences were also significant (p=0.00018; p=0.00012). It is important to note that at the time of this evaluation, the algorithm of directional microphones was enabled, which may have contributed to an improved speech perception in noisy environments, since some studies have indicated the advantages of using this algorithms for improved speech perception in noisy environments^5^ ^,^ ^18^.

These findings are extremely significant, especially when comparing the performance of OF HAs with directional microphones and OF HAs to omnidirectional microphones. The literature shows that speech recognition performance in noisy environments using OF devices with omnidirectional microphones resembles the performance unaided. In order to ensure that this variant of the HA has a benefit, it is essential to use a directional microphone^18^.

Differences between HINT scores unaided and aided have been clear (comparing situations A to B and A to C), but the results immediately after the fitting and those obtained after 90 days (situation B to C) were very similar for silence. These differences were 1.67 dB and 0.59 dB of noise. Considering the standard deviation for the Portuguese HINT^1^ of 1.7 dB for silence and 1.2 dB for noise, it is not possible to say that there has been an improvement or worsening in the results.

The evaluation of QoL has shown significant improvements in all domains after HA fitting. An improvement in social relations was expected, since individuals who are able to listen better are consequently better socializers, participate more actively in groups, and avoid social isolation caused by hearing loss^12^ ^,^ ^16^.

Some researches demonstrated improvement only in the psychological domain. These findings could be explained as a result of the short duration of use of HAs, since the re-evaluation questionnaire has been conducted just 1 month after the fitting, and the individual might have been in the acclimatization process during this period^16^.

Data from this study demonstrate that the smallest change after HA fitting occurred in the physical domain. This can be justified by the mean age of the subjects evaluated (61.96 years) involving issues related to pain, discomfort, energy, fatigue, sleep, resting, mobility, activities of daily life, dependence on medication or treatment, and ability to work^10^.

In the literature, few papers have related QoL with hearing loss using the WHOQOL-BREF. Some studies have investigated the relationship among QoL, age, sex, and the presence of hearing impairment, and found that these factors were not associated with hearing impairment^10^ ^,^ ^17^.

When we compared the results between the HINT and WHOQOL-BREF in situation A, there was no significant relationship. This can be justified by the fact that the individuals selected for this study showed a slope hearing loss with hearing preserved to 1 kHz, and in most cases, individuals with this audiometric configuration demonstrate good speech recognition performance in quiet situations^9^. In addition, the individuals evaluated had moderate losses at high frequencies, with an average threshold at 8 kHz of around 60 dBNA, which contributes to better speech recognition.

Comparing the results between the HINT and WHOQOL-BREF in situation C, a significant relationship, only between HINT noise and the domain of social relations, has been observed. These results are significant because they demonstrate that individuals with better speech perception in noisy environments have a better QoL regarding social relations after HA fitting.

This is important in this population, since individuals with hearing loss limited to high frequencies mention little difficulty in understanding speech in quiet environments, while in adverse conditions, such as when speech is distorted or in the presence of noise, the person may have many difficulties in speech intelligibility because the number of auditory cues drops considerably^8^.

There are no studies in the literature relating speech perception with QoL. The results of this study allow the conclusion that an open-fit HA improve the speech perception of individuals with hearing loss at high frequencies, both in quiet situations and in competitive noise.

The HA fitting improves the QoL of individuals in all areas assessed by the WHOQOL-BREF. Individuals with better speech recognition in noisy environments who use HAs have a better QoL.

According to these findings, this research demonstrates the importance of conducting studies to investigate the benefits that amplification can provide for individuals with slope hearing loss, thus improving the decision-making process regarding the prescription of HAs.

## CONCLUSIONS

The acoustic stimulation in hearing loss in high frequencies through the use of OF HA favors the speech recognition and improves the QoL of individuals especially in the social relations domain.
